# Comparison of Whole Plastome Sequences between Thermogenic Skunk Cabbage *Symplocarpus renifolius* and Nonthermogenic *S. nipponicus* (Orontioideae; Araceae) in East Asia

**DOI:** 10.3390/ijms20194678

**Published:** 2019-09-20

**Authors:** Seon-Hee Kim, JiYoung Yang, Jongsun Park, Takayuki Yamada, Masayuki Maki, Seung-Chul Kim

**Affiliations:** 1 Department of Biological Sciences, Sungkyunkwan University, Suwon, Gyeonggi-do 16419, Korea; desfilles@naver.com; 2 Research Institute for Dok-do and Ulleung-do Island, Department of Biology, Kyungpook National University, Daegu, Gyeongsangbuk-do 41566, Korea; whity@daum.net; 3 InfoBoss Co. Ltd., Seoul 06088, Korea; starflr@infoboss.co.kr; 4 Botanical Gardens, Tohoku University, Sendai 980-0862, Japan; net.taka.yamad@gmail.com (T.Y.); maki@m.tohoku.ac.jp (M.M.)

**Keywords:** *Symplocarpus renifolius*, *Symplocarpus nipponicus*, proto-Araceae, plastome, thermogenesis, SSRs, phylogenetic analysis

## Abstract

*Symplocarpus*, a skunk cabbage genus, includes two sister groups, which are drastically different in life history traits and thermogenesis, as follows: The nonthermogenic summer flowering *S. nipponicus* and thermogenic early spring flowering *S. renifolius*. Although the molecular basis of thermogenesis and complete chloroplast genome (plastome) of thermogenic *S. renifolius* have been well characterized, very little is known for that of *S. nipponicus*. We sequenced the complete plastomes of *S. nipponicus* sampled from Japan and Korea and compared them with that of *S. renifolius* sampled from Korea. The nonthermogenic *S. nipponicus* plastomes from Japan and Korea had 158,322 and 158,508 base pairs, respectively, which were slightly shorter than the thermogenic plastome of *S. renifolius*. No structural or content rearrangements between the species pairs were found. Six highly variable noncoding regions (*psbC/trnS*, *petA/psbJ*, *trnS/trnG*, *trnC/petN*, *ycf4/cemA*, and *rpl3/rpl22*) were identified between *S. nipponicus* and *S. renifolius* and 14 hot-spot regions were also identified at the subfamily level. We found a similar total number of SSR (simple sequence repeat) motifs in two accessions of *S. nipponicus* sampled from Japan and Korea. Phylogenetic analysis supported the basal position of subfamily Orontioideae and the monophyly of genus *Symplocarpus*, and also revealed an unexpected evolutionary relationship between *S. nipponicus* and *S. renifolius*.

## 1. Introduction

The skunk cabbage genus *Symplocarpus* Salisb. ex W.P.C.Barton belongs to the basal lineage known as Proto-Araceae of the arum family Araceae. It specifically belongs to the subfamily Orontioideae, which includes two other genera, *Orontium* L. and *Lysichiton* Schott [1,2]. While the monotypic genus *Orontium* occurs exclusively in the eastern United States, two genera, *Lysichiton* and *Symplocarpus,* are found in both eastern Asia (EAS) and western North America (WNA)/eastern North America (ENA), making these two genera an ideal system to study intercontinental disjunction in the Northern Hemisphere. In particular, genus *Symplocarpus* is of great interest for pollination biology and biogeography given its distribution pattern and thermogenesis that is characteristic of certain species. It includes five species (*S. renifolius* Schott ex Tzvelev, *S. nipponicus* Makino, *S. foetidu* (L.) Salisb. ex W.P.C.Barton, *S. nabekuraensis* Otsuka & K.Inoue, and *S. egorovii* N.S.Pavlova & V.A.Nechaev) and usually occurs in wet places and forest swamps [1,3,4]. *Symplocarpus foetidus* is the only species widely distributed in ENA and the remaining four species are found in EAS. Of the five species, *S. renifolius* and *S. foetidus* share flowering time in the early spring before the emergence of leaves and fruit ripening in the fall of the same year. The two species, especially the eastern Asian skunk cabbage *S. renifolius*, have been a subject of intensive study in thermogenesis. Blooming in early spring, often under snow, the plants can maintain a spadix temperature above 20 °C, despite ambient temperatures down to −14 °C [5,6,7,8,9,10]. Thermogenesis by flowers occurs convergently in several lineages of ancient seed plants (e.g., Araceae, Annonaceae, Cycadaceae, Magnoliaceae, Nelumbonaceae) and is known to be a direct energy reward for insect visitors by enhancing their activities [11,12,13]. Unlike thermogenic *S. renifolius*, its sister species, *S. nipponicus*, is not thermogenic and has a more spherical spadix while lacking precocious flowering. Its flowering season is in summer and its fruits ripen in the following spring [14]. Therefore, these two species have a sister relationship and demonstrate drastically different life history traits and thermogenesis during flowering. The phylogeny of *Symplocarpus* suggested that *S. nipponicus* is the earliest divergent lineage within the genus [1,15,16]. The chromosome numbers are also known for *S. foetidus* (2*n* = 4*x* = 60, tetraploid) and *S. nipponicus* (2*n* = 2*x* = 30, diploid), with a basic chromosome number of *x* = 15 [17]. The cytological evidence suggests that thermogenesis of *S. renifolius* was most likely evolved during or after polyploidization of nonthermogenic *S. nipponicus*. The divergence between *S. nipponicus* and *S. renifolius* was estimated to be 20.65 ± 6.44 million years ago (mya) based on the Bayesian molecular dating method [1].

Since the first whole chloroplast (cp) genome sequence was reported in the late 1980s, hundreds of plastomes have been characterized and utilized at various levels for phylogenetic analyses as well as DNA barcoding studies [18,19,20]. Typical angiosperm plastomes are 107–218 kilobase (kb) in length and contain 110–130 genes, with ~80 protein-coding genes, four rRNA-coding genes, and 30 tRNA-coding genes [21,22,23]. Several mutational hotspot regions, as well as some major and minor structural changes, were identified and utilized for molecular phylogenetic studies, providing insights into the molecular evolution of plastomes as well as taxonomic inferences [24,25,26,27]. Despite the taxonomic and biogeographic importance and the size of the Araceae family (117 genera and ca. 3790 species) [28], very few complete plastome sequences were assembled [29,30,31,32,33,34]. Furthermore, insufficient knowledge exists regarding the whole chloroplast genome sequences of the most basal lineage of Araceae, i.e., Proto-Araceae (including the two subfamilies Gymnostachydoideae and Orontioideae). One particular study of interest identified the complete cp genome sequence of *S. renifolius* sampled from Korea [35]. This study assembled the first cp genome of *Symplocarpus* and compared it to those of other Araceae species. 

In the present study, we determined the complete plastome sequences of nonthermogenic eastern Asian *S. nipponicus* sampled from Japan and Korea and compared them with that of its thermogenic sister species *S. renifolius*, sampled from Korea. By comparing the thermogenic and nonthermogenic types of plastome sequences, we identified changes in the organization and structure of the cp genome between the two sister species, which diverged during the early Miocene and have drastically different life history traits and thermogenesis during flowering. In addition, we aimed to identify mutational hotspot regions and plastome-based simple sequence repeat (SSR) markers, which can be useful for improving the resolution of phylogenetic relationships at lower taxonomic levels.

## 2. Results and Discussion

### 2.1. Genome Size and Features

**Figure 1:**
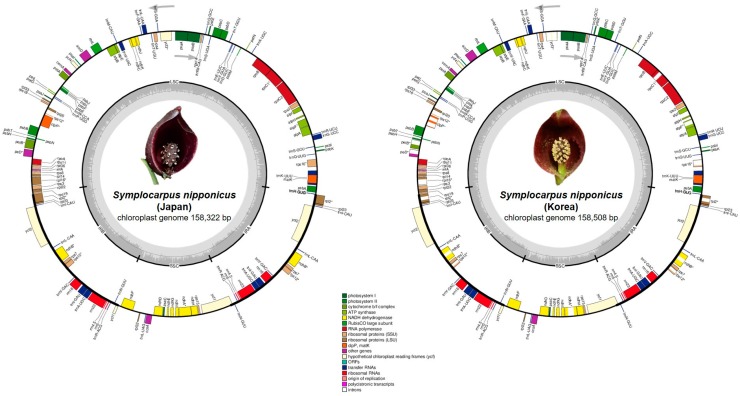
Complete plastome map of *Symplocarpus nipponicus* sampled from Korea and Japan. The genes inside and outside of the circle are transcribed in the clockwise and counterclockwise direction, repectively. Genes belonging to differenct functional groups are shown in different colors. The thick lines indicate the extent of the interted repeats (IR_A_ and IR_B_) that separate the genomes into small single copy (SSC) and large single copy (LSC) regions.

The total plastome length of *Symplocarpus nipponicus* sampled from Japan was 158,322 base pairs (bp), with a large single copy (LSC) region of 86,433 bp, small single copy (SSC) region of 20,271 bp, and two inverted repeat (IR) regions (IR_A_ and IR_B_) of 25,809 bp each. *Symplocarpus nipponicus* sampled from Korea was slightly longer, a total of 158,508 bp, with large single copy (LSC) region of 86,595bp, small single copy (SSC) region of 20,309 bp, and two inverted repeat (IR) regions of 25,802 bp (Figure 1, Table 1).

The two plastomes of *S. nipponicus* sampled from Japan and Korea contained an overall GC content of 37.4% (LSC, 34.9%; SSC, 31.0%; IRs, 43.8%) and 37.3% (LSC, 35.6%; SSC, 30.9; IR, 42.8%), respectively, and contained 130 genes, including 85 protein-coding, eight rRNA, and 37 tRNA genes (Figure 1). The complete plastome sequences of *S*. *nipponicus* sampled from Korea and Japan were nearly identical (a total of 158,104 bp identical sites; 99.7% similarity) and the *S*. *nipponicus* plastome sequence from Korea was 186 bp longer than that of *S*. *nipponicus* from Japan. The complete plastome sequences of *S*. *nipponicus* from Japan and *S*. *renifolius* from Korea showed 99.7% similarity (with a total of 158,130 bp identical sites) and the thermogenic *S*. *renifolius* plastome sequence from Korea was 199 bp longer than that of nonthermogenic *S*. *nipponicus* from Japan (Table 1).

A total of 17 genes were duplicated in the inverted repeat regions, including seven tRNA genes (*trnN-GUU*, *trnR-ACG*, *trnA-UGC*, *trnI-UUC*, *trnV-GAC*, *trnL-CAA*, and *trnI-CAU*), four rRNA genes (*5S rRNA*, *4.5S rRNA*, *23S rRNA*, and *16S rRNA*), and six protein-coding genes (*rps12*, *rps7*, *ndhB*, *ycf2*, *rpl23*, and *rpl2*). Fifteen genes (*ndhA*, *ndhB*, *petB*, *petD*, *rpl2*, *rpl16*, *rpoC1*, *rps12*, *rps16*, *trnA-UGC*, *trnG*-*UCC*, *trnI-UUC*, *trnK-UUU*, *trnL-UAA*, and *trnV-AUA*) contained one intron, while *clpP* and *ycf3* each contained two introns (Supplementary Table S1). In addition, the complete *ycf1* gene was located in the IR region at the SSC/IR_A_ junction.

The complete chloroplast genome of *S. nipponicus* was compared to ten available plastomes of Araceae (*Colocasia*, *Dieffenbachia*, *Lemna*, *Spirodela*, *Wolffiella*, *Spathiphyllum*, *Zantedeschia*, *Pinellia*, *Epipremnum*, and *Wolffia*) and *S. nipponicus* contained the smallest genome within the family [35]. The genus *Symplocarpus* represents the earliest diverged lineage within the family, i.e., the proto-Araceae, followed by sequential divergence of Lemnoideae (*Spirodela*, *Lemna*, and *Wolffia*) and Aroideae (*Dieffenbachia* and *Colocasia*) [28,36]. Given the cp genome size variations ranging from *Symplocarpus nipponicus* (158,322–158,508 bp) to *Spirodela polyrhiza* (168,788 bp) found in Araceae, any general trend in either an increase or decrease in genome size could not be postulated. Within proto-Araceae, genus *Symplocarpus* contained relatively smaller cp genome sizes compared to other members such as *Gymnostachys* and *Orontium* [37]. It is yet to be determined whether the plastome size is reduced relative to basic chromosome number in proto-Araceae. Furthermore, any plastome size differences and trends between diploids (2*n* = 2*x* = 30; *S. nipponicus*) and polyploids (2*n* = 4*x* = 60; *S. renifolius*, *S. foetidus*) within *Symplocarpus* require further study. Finally, it has been shown that the functional gene *infA* is highly variable as a pseudogene or missing gene within the family Araceae. However, like *S. renifolius*, *infA* of *S. nipponicus* was located in the LSC as an intact gene. In addition, *psbZ* was located between *trnS-UGS* and *trnG-UCC* in the LSC. When the two plastomes of thermogenic *S. renifolius* and nonthermogenic *S. nipponicus* were compared, the average value of nucleotide diversity (PI) was 0.00054, and six highly variable regions based on a significantly higher PI values of > 0.004 were identified. The regions that varied the most between *S*. *nipponicus* and *S*. *renifolius* were *psbC/trnS* and *petA/psbJ* intergenic regions, with a PI value of 0.0067 and 0.005 each, respectively. Four additional regions, including *trnS/trnG*, *trnC/petN*, *ycf4/cemA*, and *rpl3/rpl22* intergenic regions were also highly variable, with a PI value of 0.00417. Future chloroplast-based phylogeographic studies would be greatly benefited by the application of these highly variable markers (Supplementary Figure S1).

The frequency of codon usage was calculated for the cp genome based on the sequences of protein coding genes and tRNA genes, which are summarized in Supplementary Table S2. The three plastomes of *Symplocarpus* (two accessions of *S. nipponicus* from Japan and Korea and one accession of *S. renifolius* from Korea) showed the same frequency of codon usage. The usage of AUG codon was especially shared with *trnfM-CAU* and *trnI-CAU*. The codon usage was biased toward a high representation of A and U at the third codon position, which was similar to the phenomenon observed in most land plant cp genes [38].

A total of 82 editing sites in 20 protein-coding genes were identified in *S. nipponicus* from Korea, while similar numbers (83 sites) were found in *S. nipponicus* from Japan (Supplementary Table S3). The *ndhB* gene had the highest number of potential editing sites (11), followed by the *ndhD* gene (8). In accordance with previous reports [39,40], we observed that most conversions at the codon positions changed from serine (S) to leucine (L) and most RNA editing sites led to amino acid changes from polar to apolar, which resulted in an increase in protein hydrophobicity. In addition, we found one more codon position changed from serine (S) to leucine (L) of *petB* gene in *S. nipponicus* from Japan than individual from Korea.

**Figure 2:**
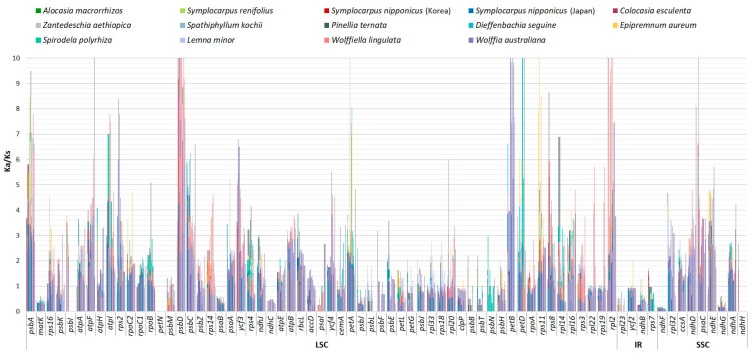
The Ka/Ks ratio of 71 protein-coding genes of cp genomes from 14 Araceae species.

Overall, the average Ka/Ks ratio of the 71 common protein-coding genes analyzed in the 14 plastomes was 0.14. For each conserved gene, the total of nine (out of 71 genes) had an average Ka/Ks ratio below one for the 14 comparison groups, suggesting that these genes were subject to strong purifying selection pressures in Araceae chloroplasts. Those genes included one photosystem I subunit gene (*psaI*), two photosystem II subunit gene (*psbB* and *psbM*), three NADP dehydrogenase (*ndhC*, *ndhF*, and *ndhG*), one ribosome large subunit gene (*rpl23*), and one cytochrome c biogenesis maturase (*matK*). However, the majority (62 of 71 genes) had a Ka/Ks ratio of > 1, which suggests that these genes were positively selected within the Araceae family. *Alocasia macrorhizos, Lemna minor*, and *Woffia australiana* showed a high value of Ka/Ks > 1 in all 14 taxa comparison. In general, previous studies showed that Ka/Ks values are usually less than one [41] because synonymous nucleotide substitutions occur more frequently than nonsynonymous substitutions. Surprisingly, our results in the Araceae family suggested that most protein-coding genes underwent positive selection pressures (Figure 2).

### 2.2. Microsatellites Analysis

**Figure 3:**
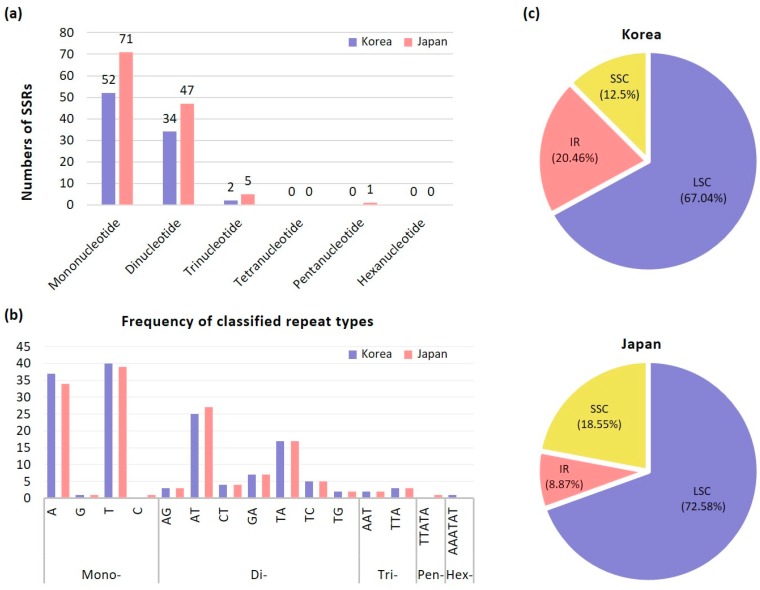
Analyses of repeated sequences in two accessions of *Symplocarpus nipponicus* plastome. (**a**) Numbers of different repeat types; (**b**) Numbers of identified SSRs motifs in different repeat class types; (**c**) Frequency of repeat types in LSC, SSC, and IR regions.

We found similar number of SSR motifs between two accessions of *S. nipponicus* sampled from Japan and Korea. Of a total of 124 unique consensus SSRs identified from *S*. *nipponicus* in Japan (Supplementary Table S4), 90 (72.58%) were located in the LSC region, 23 (18.55%) in the SSC region, and 11 (8.87%) in the two IR regions (Figure 3). Most of the SSRs were located in intergenic regions (*n* = 71; 57.3%), whereas 22 (17.7%) were located in introns, 26 (21%) in protein coding genes, and 5 (4%) in tRNA and rRNA genes (*trnI*, *trnS* and *23S rRNA*, respectively). Therefore, 75% of the SSRs were located in intergenic and intron regions, while only 25% were distributed in conserved genic regions. In addition, mononucleotide, dinucleotide, trinucleotide, and pentanucleotide repeats were detected in 71 (57.3%), 47 (37.9%), five (4%), and one (0.8%) unique consensus SSRs, respectively. No tetranucleotides and hexanucleotides were found. Of a total of 88 unique consensus SSRs identified from *S*. *nipponicus* in Korea (Supplementary Table S5), 59 (67.04%) were located in the LSC region, 11 (12.5%) in the SSC region, and 18 (20.46%) in the two IR regions.

Most of the SSRs were located in intergenic regions (*n* = 56; 63.6%), whereas 14 (15.9%) were located in introns, 15 (17.1%) in protein coding genes, and three (3.4%) in *23S rRNA* genes. Therefore, 79.5% of the SSRs were located in intergenic and intron regions, while only 20.5% were distributed in conserved genic regions. In addition, mononucleotide, dinucleotide, and trinucleotide repeats were detected in 52 (59.09%), 34 (38.63%), and two (2.27%) unique consensus SSRs, respectively. No tetranucleotide, pentanucleotide, and hexanucleotides were found. Given the similar parameter settings of the MISA program to identify SSR motifs, we found slightly higher numbers of SSRs in the LSC region in *S. nipponicus* from Japan (90 SSRs; 72.58%) than in that of *S. renifolius* (85 SSRs; 70.25%), but significantly more than *S*. *nipponicus* from Korea (59 SSRs; 67.04%) [35]. However, we found slightly fewer numbers of SSR motifs in the SSC region of *S. nipponicus* (11 and 23 SSRs in accession from Korea and Japan, respectively) than in that of *S. renifolius* (30 SSRs), but significantly higher numbers in the IR regions (18 and 11 in accession of *S. nipponicus* from Korea and Japan, respectively and six in *S. renifolius*). The total number of SSR motifs found in these two congeneric species (*Symplocarpus nipponicus* and *S. renifolius*) was significantly higher than that in the members of Lemnoideae (66 in *Wolffia*, 77 in *Wolffiella*, 85 in *Spirodela*, and 71 in *Lemna*) [35]. It appears that there is no correlation between the plastome size and the total number of SSRs. In addition to highly variable cp markers for phylogeographic studies, cp SSR markers found in nonthermogenic *S. nipponicus* can be useful for future population-level studies of individual species or for comparative population genetic studies between intercontinental disjunct species pairs in eastern Asia (*S. renifolius* and *S. nipponicus*) and in eastern North America (*S. foetidus*).

### 2.3. Comparative Analysis of Genome Structure

**Figure 4:**
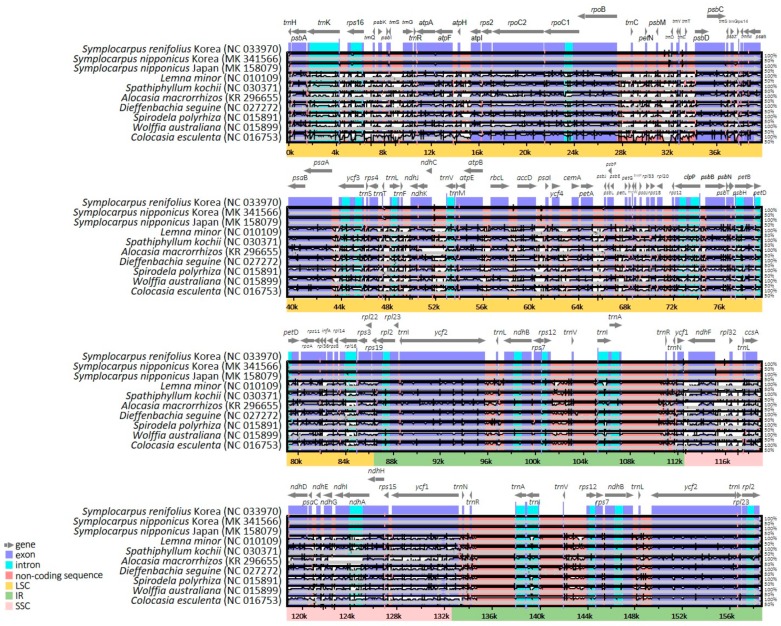
Alignment visualization of chloroplast genome sequences from nine representative species of Araceae. VISTA-based identity plots revealed the sequence identity of ten chloroplast genomes relative to the reference *Symplocarpus renifolius*. Vertical scale indicates the percent identity, from 50 to 100%. Coding and noncoding regions are in blue and pink, respectively. Gray arrows above the alignment indicate the position and direction of each gene.

The complete plastome sequences of two accessions of *Symplocarpus nipponicus*, *S. renifolius*, *Lemna minor*, *Spathiphyllum kochii*, *Alocasia macrorrhizos*, *Dieffenbachia seguine*, *Spirodela polyrhiza*, *Wolffia australiana*, and *Colocasia esculenta* were plotted using mVISTA analysis, using annotated *S. renifolius* plastome as a reference (Figure 4). Based on the overall sequence identity, indicated by peaks and valleys among all nine species (ten accessions) of Araceae, the results indicated that the LSC region was most divergent, that the two IR regions were highly conserved, and that the non-coding regions were more divergent and variable than the coding regions. 

**Figure 5:**
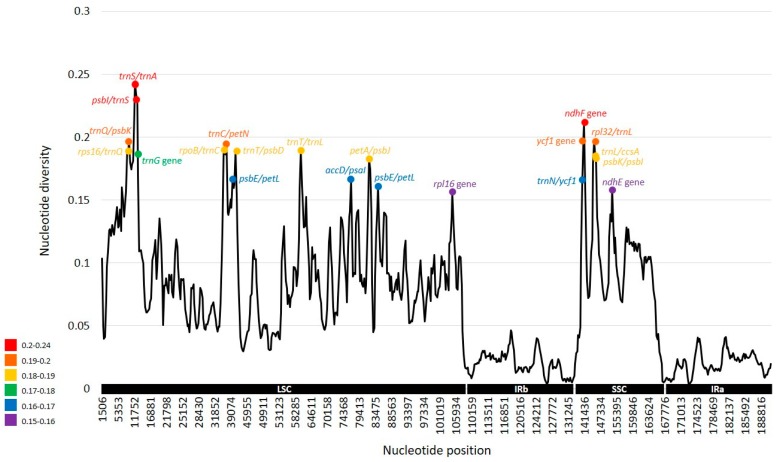
Sliding window analysis of the whole chloroplast genomes of nine Araceae species.

Sliding window analysis revealed highly variable regions in the Araceae plastome (Figure 5). The average value of nucleotide diversity (PI) over the entire plastome was 0.07044 and 14 highly variable regions based on a significantly higher PI value of > 0.17 were identified. The most variable of these was the *trnS/trnA* intergenic region (PI = 0.24358), followed by the other two regions, *psbI/trnS* (PI = 0.21919) and *ndhF* gene (PI = 0.2105). Other variable regions were also identified, including *ycf*1 gene (PI = 0.19811) and ten intergenic regions (*trnQ/psbK* (PI = 0.19756), *rpl32/trnL* (PI = 0.19697), *trnC/petN* (PI = 0.19256), *trnT/trnL* (PI = 0.18911), *rps*16/*trnQ* (PI = 0.18875), *trnT*/*psbD* (PI = 0.18861), *rpoB/trnC* (PI = 0.18628), *trnL/ccsA* (PI = 0.18428), *psbK/psbI* (PI = 0.18322), and *petA/psbJ* (PI = 0.1825)). When these hotspot regions based on the representative lineages within the family Araceae were compared to those from two congeneric species of *Symplocarpus*, we found that highly variable regions based on the family level are not necessarily the same as those of within the genus level *Symplocarpus*, with the exception of *petA/psbJ* and *trnC/petN* regions. Compared to highly variable noncoding cp regions identified by Shaw et al. [42,43], we found that five regions (*rpl32/trnL*, *trnT/trnL*, *rps16/trnQ*, *trnT/psbD*, and *petA/psbJ*) in Araceae are consistently and highly variable regardless of taxonomic level. The remaining nine regions in Araceae were not identified as hotspot regions in angiosperms, suggesting no universal “best” region. Overall, we identified several highly variable plastome regions in Araceae, which may help resolve phylogenetic relationships and could potentially be used as effective markers for barcoding and phylogenetic studies of higher taxonomic levels. 

### 2.4. Phylogenetic Analysis

**Figure 6:**
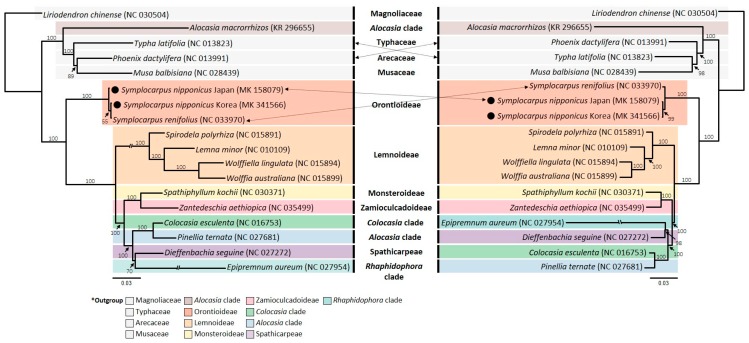
Phylogenetic tree reconstruction of 18 taxa using maximum likelihood based on datasets of the entire chloroplast genome sequences (**left**) and shared 85 coding genes only (**right**). ML topology was shown with ML bootstrap support (BS) value given at each node.

The ML (maximum likelihood) tree confirmed previously known phylogenetic relationships within the family Araceae based on earlier studies, while unexpected relationships and positions of certain taxa were also revealed in this study [28,36,44,45,46] (Figure 6). Furthermore, some topological incongruences were found between the entire plastome sequence data set and the 85 concatenated coding gene data set. Reconfirmation of previously known relationships included (1) early divergence of *Symplocarpus* (subfamily Orontioideae) within Araceae, (2) the monophyly of Lemnoideae and its sister relationship to the rest of Araceae, (3) relationships among four genera of Lemnoideae, and (4) the close relationship between *Colocasia* and *Pinellia*. Few unexpected relationships based on the current phylogeny included the position of *Spathiphyllum*, *Zantedeschia*, and *Epipremnum*. Within the subfamily Monsteroideae, the previous studies suggested that *Epipremnum* (*Rhaphidophora* clade) is related to *Spathiphyllum* (Monsteroideae). However, our phylogeny suggested that *Epipremnum* is closely related to *Dieffenbachia* (Spathicarpeae). The previous studies suggested that *Dieffenbachia* is more closely related to *Zantedeschia* (*Zantedeschia* clade) than it is to *Epipremnum*. One most surprising and unexpected finding in this study was the position of *Alocasia macrorrhizos* (KR 296655). Our phylogenetic tree based on the complete cp genome sequence and 85 concatenated coding genes suggested that *Alocasia macrorrhizos* is not closely related to other members of the same family Araceae. Rather, it came out as a sister to the clade containing Musaceae, Arecaceae, and Typhaceae (Figure 6). On the other hand, several previous studies suggested that, although *Alocasia* appears to be not monophyletic, members of this genus are closely related to *Colocasia* and *Pinellia* in *Dracunculus* clade [45,46], *Pistia* clade [28,44], or *Ambrosina* clade [36]. Therefore, the unusual position of *Alocasia* out of its typical position within Araceae was highly exceptional and unexpected. It has been well recognized that both analytical (e.g., the choice of optimality criterion, taxon sampling, specific assumptions in the modeling of sequence evolution, etc.) and biological factors (e.g., violations of the orthology due to lineage sorting, paralogy, and horizontal gene transfer, character sampling bias related to the length of the genes, systematic error due to the presence of a nonphylogenetic signal in the data, etc.) cause phylogenetic incongruence (see Reference [47] and references therein). With regard to the position of *Alocasia macrorrhizos*, complete plastid sequence (KR 296655) outside of its own family Araceae, it may be caused by either long branch attraction or simply a taxon misidentification issue. It appears that *Alocasia macrorrhizos* (KR 296655) shows somewhat longer branch length compared to that of other lineages, but other factors (e.g., optimality criteria, molecular model assumptions, chloroplast capture, etc.) also likely contributed to its unusual position. The optimality criteria as a contributing factor, however, can be ruled out given the same tree topology found between the ML and BI (Bayesian Inference) tree (with unspecified model selection) based on the complete plastid sequences of Alismatales [34]. It is also possible that misidentification of taxon or potential mistakes during sampling and sequencing acquisition processes likely caused its unusual position. Since only one species for the complete chloroplast genome of *Alocasia* has been sequenced and reported, we could not confirm any of these possibilities [32,34]. It remains to be confirmed as to what factors contributed to unusual position of *Alocasia macrorrhizos* by sampling additional species of *Alocasia*. 

Within the genus *Symplocarpus*, we found that *S. nipponicus* is sister to the clade containing *S. foetidus* and *S. renifolius*, revealing overall phylogenetic relationships among the three common species [48,49]. As suggested previously in Lee et al. [48], one important phylogenetic incongruence based on the complete plastome and 85 coding genes data set is the differing position of the two nonthermogenic conspecific *S. nipponicus* accessions (Figure 6). In this study, we sequenced the two accessions of *S. nipponicus* sampled from Korea and Japan. The complete plastid sequence phylogeny showed that *S. nipponicus* sampled from Korea is more closely related to *S. renifolius* sampled from Korea than to its conspecific population sampled from Japan (55% BS support). On the other hand, the 85 concatenated coding genes data set suggested that two accessions of *S. nipponicus* are sister to each other (99% BS support). The complete plastid sequence-based phylogeny was unexpected given drastically different morphological, cytological, and life history traits between thermogenic *S. renifolius* and nonthermogenic *S. nipponicus*. However, our broader phylogenetic study based on extensive sampling of proto-Araceae found that *S. renifolius* in Korea is diploid, unlike known tetraploid conspecific populations in Japan, and that populations of *S. renifolius* in Korea are more closely related to *S. nipponicus* in Korea than to conspecific populations in Japan [48]. In addition, Korean populations of *S. renifolius* are morphologically distinctive from those of Japan, warranting new taxonomic recognition. Therefore, the relationship suggested based on the complete plastid sequence in this study is not completely unexpected, perhaps reflecting true evolutionary relationship among those taxa. It requires further confirmation based on different types of molecular markers, especially from nuclear single-copy or low-copy genes.

## 3. Materials and Methods

### 3.1. Plastome Sequencing, Assembly, and Annotation

Fresh *Symplocarpus nipponicus* leaves from a single plant were collected from the natural population in Japan (voucher specimen: SKKU_Kim 1608001) and Korea (voucher specimens: SKKU_Kim et al., 160711001). Total DNA was isolated using the DNeasy plant Mini Kit (Qiagen, Carlsbad, CA, USA) and sequenced using the Illumina HiSeq 4000 (Illumina, Inc., San Diego, CA, USA) at Macrogen Corporation (Seoul, Korea). The resulting paired-end reads were assembled de novo using Velvet v. 1.2.10 with multiple k-mers [50]. The tRNAs were confirmed using tRNAscan-SE [51]. Annotation was conducted using Geneious R10 [52] and the annotated plastome sequences were submitted to GenBank (accession number MK158079 from Japan and MK341566 from Korea). The annotated GenBank format sequence files were used to draw the circular maps using OGDRAW program v1.2 [53].

### 3.2. Comparative Plastome Analysis

The complete plastome of nonthermogenic *Symplocarpus nipponicus* sampled from Japan and Korea was compared to that of *S. renifolius* (NC 003970), a thermogenic sister species, using mVISTA [54] in Shuffle-LAGAN mode [55]. The two accessions of *S. nipponicus* and *S. renifolius* cp genome sequences and eight additionally related plastome (*Lemna*, *Wolffia*, *Wolffiella*, *Spirodela*, *Spathiphyllum*, *Dieffenbachia*, *Alocasia*, and *Colocasia*) sequences were aligned using MAFFT v. 7 [56] and adjusted manually using Geneious R10 [52]. DnaSP v. 6.10 software [57] using a sliding window analysis with a step size of 200 bp and a window length of 800 bp was carried out to determine the nucleotide diversity (PI) of the plastome. The codon usage frequency was calculated by using MEGA7 [58] with relative synonymous codon usage (RSCU) value, which is a simple measure of non-uniform usage of synonymous codons in a coding sequence [59]. To predict the possible RNA editing sites in *S. nipponicus* from Korea and Japan, protein-coding genes were conducted using the online program predictive RNA editor for plants (PREP) suite [60] with 22 genes as reference, based on cut off value of 0.8. Analyses based on the complete cp genomes and concatenated sequences of 71 common protein-coding genes among the studied species were conducted by MAFFT v. 7 [56], using Geneious R10 [52]. Using DnaSP v. 6.10 software [57], we calculated the Ka/Ks ratios of the three *Symplocarpus* cp genomes (*S. renifolius* and *S. nipponicus* from Korea and *S. nipponicus* from Japan) compared to 11 closely related species in the family of Araceae (i.e., Lemnoideae, Monsteroideae, Zamioculcadoideae, and Spathicarpeae).

### 3.3. Tandem Repeat and Microsatellite Analysis

Simple sequence repeat (SSR) markers were identified in the plastome sequence using MISA [61] with minimum repeat thresholds of 10 for mononucleotide repeats, four for dinucleotide repeats, four for trinucleotide repeats, four for tetranucleotide repeats, four for pentanucleotide repeats, and three for hexanucleotide repeats. 

### 3.4. Phylogenetic Analysis

For the phylogenetic analysis, 17 representative species from the Mesangiospermae (13 species from Araceae in Alismatales of Monocots, three from representative orders of Monocots (one species from Musaceae of Zingiberales, one species from Arecaceae of Arecales, and one species from Typhaceae of Poales), and one species from Magnoliaceae in Magnoliids) were aligned using MAFFT v. 7 in Geneious [52]. Maximum likelihood (ML) analysis based on the best-fit model of GTR+F+R4 was conducted using IQ-TREE v. 1.4.2 [62]. *Liriodendron chinense* (Magnoliids) was used as the outgroup taxon and nonparametric bootstrap analysis was performed with 1000 replicates. 

## 4. Conclusions

We determined the complete plastome sequence of nonthermogenic *Symplocarpus nipponicus* sampled from Japan and Korea and compared it to the plastomes of thermogenic sister species *S*. *renifolius* and several other representative species from two subfamilies (Lemnoideae and Aroideae) of Araceae. Comparative phylogenomic analysis between thermogenic and nonthermogenic *Symplocarpus* sister species revealed highly conserved plastomes despite drastically different life history traits and thermogenesis. We found six highly variable regions that could be used as powerful molecular markers for future phylogeographic and population genetic studies (e.g., Reference [49]). Moreover, comparative phylogenomic analysis with five additional species from Lemnoideae (*Lemna*), Aroideae (*Colocasia*), Mosteroideae, Zamioculcadoieae, and Spathicarpeae, revealed 14 highly variable regions with high PI values (*trnS/trnA*, *psbI/trnS, ndhF* gene, *ycf1* gene, *trnQ/psbK, rpl32/trnL, trnC/petN, trnT/trnL*, *rps16/trnQ, trnT/psbD*, *rpoB/trnC*, *trnL/ccsA, psbK/psbI*, and *petA/psbJ)*. These mutation hotspots could be used as useful markers to improve the resolution of unresolved phylogenetic relationships within Araceae as well as effective barcoding markers for the identification of species.

## Figures and Tables

**Table 1:** Summary statistics of two accessions of *Symplocarpus nipponicus* and *S. renifolius* plastomes.

Taxa	*S. nipponicus* (Korea)	*S. nipponicus* (Japan)	*S. renifolius* (Korea)
Accession Number	MK341566	MK158079	KY039276
Total cpDNA size (bp)/GC content (%)	158,508/37.3	158,322/37.4	158,521/37.3
LSC size (bp)/GC content (%)	86,595/35.6	86,433/34.9	86,620/35.6
IR size (bp)/GC content (%)	25,802/42.8	25,809/43.8	25,801/42.9
SSC size (bp)/GC content (%)	20,309/30.9	20,271/31.0	20,299/31.0
Number of genes	130	130	130
Number of protein-coding genes	85	85	85
Number of tRNA genes	37	37	37
Number of rRNA genes	8	8	8
Number of duplicated genes	17	17	17

## Supplementary Materials

The following are available online at https://www.mdpi.com/1422-0067/20/19/4678/s1, Figure S1. Sliding window analysis of the whole chloroplast genomes between *Symplocarpus nipponicus* and *S. renifolius*. Table S1. Genes with introns in the two accessions of *Symplocarpus nipponicus* chloroplast genome and the length of the exons and introns. Table S2. Codon-anticodon recognition pattern and codon usage for *Symplocarpus* chloroplast genomes (two accessions of *S. nipponicus* and one accession of *S. renifolius*). RSCU = relative synonymous codon usage. Table S3. Predicted RNA editing sites in the cp genome of two accessions of *S. nipponicus* from Japan and Korea. Table S4. Repeat sequences and their distribution in the *Symplocarpus nipponicus* chloroplast genome in Japan. Table S5. Repeat sequences and their distribution in the *Symplocarpus nipponicus* chloroplast genome in Korea. Supplementary Data S1. The data matrix of 12 representative plastomes of Mesangiospermae.
