# Supplementary material for: Comparison of Whole Plastome Sequences between Thermogenic Skunk Cabbage Symplocarpus renifolius and Nonthermogenic S. nipponicus (Orontioideae; Araceae) in East Asia
Source: Int J Mol Sci. 2019 Sep 20;20(19):4678. doi: 10.3390/ijms20194678 (PMC6801674; doi:10.3390/ijms20194678)
Supplement: Supplementary file 1 [file ijms-20-04678-s001.zip › Table S1.docx]

**Table S1.** Genes with introns in the two accessions of *Symplocarpus nipponicus* chloroplast genome and the length of the exons and introns.

| **Location** | **Gene** | **Sample** | **exonⅠ** | **intronⅠ** | **exonⅡ** | **intron Ⅱ** | **exon Ⅲ** |
| --- | --- | --- | --- | --- | --- | --- | --- |
|  |  |  | **(bp)** | **(bp)** | **(bp)** | **(bp)** | **(bp)** |
| LSC | *rps16* | *S. nipponicus* (Japan) | 40 | 1,032 | 254 |  |  |
|  |  | *S. nipponicus* (Korea) | 40 | 1,000 | 224 |  |  |
|  | *atpF* | *S. nipponicus* (Japan) | 145 | 786 | 410 |  |  |
|  |  | *S. nipponicus* (Korea) | 145 | 786 | 410 |  |  |
|  | *rpoC1* | *S. nipponicus* (Japan) | 453 | 767 | 1,620 |  |  |
|  |  | *S. nipponicus* (Korea) | 453 | 767 | 1,620 |  |  |
|  | *ycf3* | *S. nipponicus* (Japan) | 126 | 760 | 230 | 762 | 153 |
|  |  | *S. nipponicus* (Korea) | 126 | 762 | 230 | 762 | 153 |
|  | *clpP* | *S. nipponicus* (Japan) | 71 | 792 | 292 | 682 | 246 |
|  |  | *S. nipponicus* (Korea) | 71 | 796 | 292 | 682 | 246 |
|  | *petB* | *S. nipponicus* (Japan) | 6 | 793 | 642 |  |  |
|  |  | *S. nipponicus* (Korea) | 6 | 793 | 642 |  |  |
|  | *petD* | *S. nipponicus* (Japan) | 8 | 732 | 475 |  |  |
|  |  | *S. nipponicus* (Korea) | 8 | 732 | 475 |  |  |
|  | *rpl16* | *S. nipponicus* (Japan) | 9 | 1,010 | 399 |  |  |
|  |  | *S. nipponicus* (Korea) | 9 | 1,017 | 399 |  |  |
| IR | *rpl2* | *S. nipponicus* (Japan) | 385 | 664 | 431 |  |  |
|  |  | *S. nipponicus* (Korea) | 385 | 664 | 431 |  |  |
|  | *ndhB* | *S. nipponicus* (Japan) | 777 | 700 | 756 |  |  |
|  |  | *S. nipponicus* (Korea) | 777 | 700 | 756 |  |  |
|  | *rps12* | *S. nipponicus* (Japan) | 232 | 540 | 26 |  |  |
|  |  | *S. nipponicus* (Korea) | 232 | 540 | 26 |  |  |
| SSC | *ndhA* | *S. nipponicus* (Japan) | 553 | 1,136 | 539 |  |  |
|  |  | *S. nipponicus* (Korea) | 553 | 1,133 | 539 |  |  |
