# Supplementary material for: Comparison of Whole Plastome Sequences between Thermogenic Skunk Cabbage Symplocarpus renifolius and Nonthermogenic S. nipponicus (Orontioideae; Araceae) in East Asia
Source: Int J Mol Sci. 2019 Sep 20;20(19):4678. doi: 10.3390/ijms20194678 (PMC6801674; doi:10.3390/ijms20194678)
Supplement: Supplementary file 1 [file ijms-20-04678-s001.zip › Table S2.docx]

**Table S2.** Codon-anticodon recognition pattern and codon usage for *Symplocarpus* chloroplast genomes (two accessions of *S. nipponicus* and one accession of *S. renifolius*). RSCU = relative synonymous codon usage.

| **Codon (Amino acid)** | ***S. nipponicus* (Japan)** | | **tRNA** | ***S. nipponicus* (Korea)** | | **tRNA** | ***S. renifolius* (Korea)** | | **tRNA** |
| --- | --- | --- | --- | --- | --- | --- | --- | --- | --- |
|  | **Count** | **RSCU** |  | **Count** | **RSCU** |  | **Count** | **RSCU** |  |
| UUU (F) | 926 | 1.24 | *trnK-UUU* | 928 | 1.24 | *trnK-UUU* | 921 | 1.24 | *trnK-UUU* |
| UUC (F) | 566 | 0.76 | *trnE-UUC* | 567 | 0.76 | *trnE-UUC* | 567 | 0.76 | *trnE-UUC* |
| UUA (L) | 823 | 1.82 |  | 820 | 1.82 |  | 825 | 1.83 |  |
| UUG (L) | 559 | 1.24 | *trnQ-UUG* | 559 | 1.24 | *trnQ-UUG* | 559 | 1.24 |  |
| CUU (L) | 565 | 1.25 |  | 565 | 1.25 |  | 564 | 1.25 |  |
| CUC (L) | 190 | 0.42 |  | 189 | 0.42 |  | 189 | 0.42 |  |
| CUA (L) | 377 | 0.83 |  | 377 | 0.84 |  | 378 | 0.84 |  |
| CUG (L) | 196 | 0.43 |  | 197 | 0.44 |  | 195 | 0.43 |  |
| AUU (I) | 1,099 | 1.46 |  | 1,101 | 1.46 |  | 1,102 | 1.46 |  |
| AUC (I) | 452 | 0.6 |  | 454 | 0.6 |  | 453 | 0.6 |  |
| AUA (I) | 712 | 0.94 |  | 713 | 0.94 |  | 713 | 0.94 |  |
| AUG (M) | 617 | 1 |  | 616 | 1 |  | 616 | 1 |  |
| GUU (V) | 508 | 1.41 | *trnN-GUU* | 508 | 1.41 | *trnN-GUU* | 508 | 1.41 | *trnN-GUU* |
| GUC (V) | 188 | 0.52 | *trnD-GUC* | 188 | 0.52 | *trnD-GUC* | 186 | 0.52 | *trnD-GUC* |
| GUA (V) | 530 | 1.47 | *trnY-GUA* | 529 | 1.46 | *trnY-GUA* | 531 | 1.47 | *trnY-GUA* |
| GUG (V) | 219 | 0.61 | *trnH-GUG* | 220 | 0.61 | *trnH-GUG* | 219 | 0.61 | *trnH-GUG* |
| UCU (S) | 565 | 1.61 | *trnR-UCU* | 566 | 1.61 | *trnR-UCU* | 565 | 1.61 | *trnR-UCU* |
| UCC (S) | 359 | 1.03 | *trnG-UCC* | 359 | 1.02 | *trnG-UCC* | 358 | 1.02 | *trnG-UCC* |
| UCA (S) | 453 | 1.29 |  | 454 | 1.29 |  | 454 | 1.3 |  |
| UCG (S) | 188 | 0.54 |  | 187 | 0.53 |  | 189 | 0.54 |  |
| CCU (P) | 406 | 1.47 |  | 405 | 1.47 |  | 407 | 1.48 |  |
| CCC (P) | 235 | 0.85 |  | 235 | 0.85 |  | 235 | 0.85 |  |
| CCA (P) | 331 | 1.2 | *trnW-CCA* | 331 | 1.2 | *trnW-CCA* | 331 | 1.2 | *trnW-CCA* |
| CCG (P) | 130 | 0.47 |  | 130 | 0.47 |  | 128 | 0.47 |  |
| ACU (T) | 525 | 1.56 |  | 525 | 1.56 |  | 525 | 1.56 |  |
| ACC (T) | 248 | 0.74 |  | 247 | 0.73 |  | 247 | 0.73 |  |
| ACA (T) | 419 | 1.24 |  | 419 | 1.24 |  | 419 | 1.24 |  |
| ACG (T) | 157 | 0.47 | *trnR-ACG* | 157 | 0.47 | *trnR-ACG* | 156 | 0.46 | *trnR-ACG* |
| GCU (A) | 661 | 1.88 | *trnS-GCU* | 663 | 1.88 | *trnS-GCU* | 661 | 1.88 | *trnS-GCU* |
| GCC (A) | 207 | 0.59 | *trnG-UCC* | 206 | 0.59 | *trnG-UCC* | 207 | 0.59 |  |
| GCA (A) | 398 | 1.13 | *trnC-GCA* | 398 | 1.13 | *trnC-GCA* | 398 | 1.13 | *trnC-GCA* |
| GCG (A) | 140 | 0.4 |  | 140 | 0.4 |  | 139 | 0.4 |  |
| UAU (Y) | 778 | 1.56 |  | 776 | 1.56 |  | 775 | 1.56 |  |
| UAC (Y) | 217 | 0.44 | *trnV-UAC* | 217 | 0.44 | *trnV-UAC* | 216 | 0.44 | *trnV-UAC* |
| UAA (*) | 38 | 1.33 | *trnL-UAA* | 37 | 1.29 | *trnL-UAA* | 36 | 1.26 | *trnL-UAA* |
| UAG (*) | 27 | 0.94 | *trnL-UAG* | 27 | 0.94 | *trnL-UAG* | 28 | 0.98 | *trnL-UAG* |
| CAU (H) | 506 | 1.53 | *trnM-CAU* | 506 | 1.53 | *trnI-CAU* | 507 | 1.53 | *trnI-CAU* |
| CAC (H) | 155 | 0.47 |  | 156 | 0.47 |  | 156 | 0.47 |  |
| CAA (Q) | 668 | 1.47 | *trnL-CAA* | 669 | 1.47 | *trnL-CAA* | 670 | 1.47 | *trnL-CAA* |
| CAG (Q) | 240 | 0.53 |  | 241 | 0.53 |  | 241 | 0.53 |  |
| AAU (N) | 974 | 1.53 |  | 971 | 1.53 |  | 970 | 1.53 |  |
| AAC (N) | 300 | 0.47 |  | 298 | 0.47 |  | 298 | 0.47 |  |
| AAA (K) | 1,037 | 1.47 |  | 1,036 | 1.47 |  | 1,036 | 1.47 |  |
| AAG (K) | 373 | 0.53 |  | 373 | 0.53 |  | 373 | 0.53 |  |
| GAU (D) | 888 | 1.62 | *trnI-GAU* | 888 | 1.62 | *trnI-GAU* | 889 | 1.62 | *trnI-GAU* |
| GAC (D) | 207 | 0.38 | *trnV-GAC* | 207 | 0.38 | *trnV-GAC* | 207 | 0.38 | *trnV-GAC* |
| GAA (E) | 1,013 | 1.47 | *trnF-GAA* | 1,010 | 1.47 | *trnF-GAA* | 1,009 | 1.47 | *trnF-GAA* |
| GAG (E) | 365 | 0.53 |  | 365 | 0.53 |  | 364 | 0.53 |  |
| UGU (C) | 228 | 1.48 | *trnT-UGU* | 228 | 1.48 | *trnT-UGU* | 228 | 1.48 | *trnT-UGU* |
| UGC (C) | 81 | 0.52 | *trnA-UGC* | 81 | 0.52 | *trnA-UGC* | 81 | 0.52 | *trnA-UGC* |
| UGA (*) | 21 | 0.73 | *trnS-UGA* | 22 | 0.77 | *trnS-UGA* | 22 | 0.77 | *trnS-UGA* |
| UGG (W) | 446 | 1 | *trnP-UGG* | 446 | 1 | *trnP-UGG* | 446 | 1 | *trnP-UGG* |
| CGU (R) | 369 | 1.36 |  | 369 | 1.36 |  | 369 | 1.36 |  |
| CGC (R) | 100 | 0.37 |  | 101 | 0.37 |  | 100 | 0.37 |  |
| CGA (R) | 351 | 1.29 |  | 350 | 1.29 |  | 351 | 1.29 |  |

**Table S2.** *Cont.*

| **Codon (Amino acid)** | ***S. nipponicus* (Japan)** | | **tRNA** | ***S. nipponicus* (Korea)** | | **tRNA** | ***S. renifolius* (Korea)** | | **tRNA** |
| --- | --- | --- | --- | --- | --- | --- | --- | --- | --- |
|  | **Count** | **RSCU** |  | **Count** | **RSCU** |  | **Count** | **RSCU** |  |
| CGG (R) | 113 | 0.42 |  | 113 | 0.42 |  | 113 | 0.42 |  |
| AGU (S) | 426 | 1.22 |  | 428 | 1.22 |  | 425 | 1.21 |  |
| AGC (S) | 110 | 0.31 |  | 110 | 0.31 |  | 110 | 0.31 |  |
| AGA (R) | 521 | 1.92 |  | 520 | 1.92 |  | 521 | 1.92 |  |
| AGG (R) | 173 | 0.64 |  | 173 | 0.64 |  | 173 | 0.64 |  |
| GGU (G) | 601 | 1.35 | *trnT-GGU* | 600 | 1.35 | *trnT-GGU* | 598 | 1.34 | *trnT-GGU* |
| GGC (G) | 155 | 0.35 |  | 155 | 0.35 |  | 155 | 0.35 |  |
| GGA (G) | 738 | 1.66 | *trnS-GGA* | 738 | 1.66 | *trnS-GGA* | 737 | 1.66 | *trnS-GGA* |
| GGG (G) | 288 | 0.65 |  | 287 | 0.64 |  | 289 | 0.65 |  |

Notes: Calculations were made from all coding regions combined and based on the DNA sequences of protein coding genes and tRNA genes.
