# Supplementary material for: Comparison of Whole Plastome Sequences between Thermogenic Skunk Cabbage Symplocarpus renifolius and Nonthermogenic S. nipponicus (Orontioideae; Araceae) in East Asia
Source: Int J Mol Sci. 2019 Sep 20;20(19):4678. doi: 10.3390/ijms20194678 (PMC6801674; doi:10.3390/ijms20194678)
Supplement: Supplementary file 1 [file ijms-20-04678-s001.zip › Table S3.docx]

**Table S3.** Predicted RNA editing sites in the cp genome of two accessions of *S. nipponicus* from Japan and Korea.

| **Gene** | **Nucleotide**  **Position** | **Amino Acid**  **Position** | **Codon**  **Conversion** | **Score** | **Nucleotide**  **Position** | **Amino Acid**  **Position** | **Codon**  **Conversion** | **Score** | |
| --- | --- | --- | --- | --- | --- | --- | --- | --- | --- |
|  | *Symplocarpus nipponicus* (Japan) | | | | *Symplocarpus nipponicus* (Korea) | | | | |
| *matK* | 907 | 303 | CAC (H) =>  TAC (Y) | 1 | 907 | 303 | CAC (H) =>  TAC (Y) | | 1 |
|  | 1,198 | 400 | CCA (P) =>  TCA (S) | 0.86 | 1,198 | 400 | CCA (P) =>  TCA (S) | | 0.86 |
|  | 1,255 | 419 | CAC (H) =>  TAC (Y) | 1 | 1,255 | 419 | CAC (H) =>  TAC (Y) | | 1 |
|  | 1,328 | 443 | TCG (S) =>  TTG (L) | 1 | 1,328 | 443 | TCG (S) =>  TTG (L) | | 1 |
| *accD* | 809 | 270 | TCG (S) => | 0.8 | 809 | 270 | TCG (S) => TTG (L) | | 0.8 |
|  |  |  | TTG (L) |  |  |  |  |  |  |
|  | 1,172 | 391 | TCA (S) => | 1 | 1,172 | 391 | TCA (S) => TTA (L) | | 1 |
|  |  |  | TTA (L) |  |  |  |  |  |  |
|  | 1,376 | 459 | TCA (S) => | 1 | 1,376 | 459 | TCA (S) => TTA (L) | | 1 |
|  |  |  | TTA (L) |  |  |  |  |  |  |
|  | 1,418 | 473 | CCT (P) => | 1 | 1,418 | 473 | CCT (P) => CTT (L) | | 1 |
|  |  |  | CTT (L) |  |  |  |  |  |  |
| *atpA* | 773 | 258 | TCA (S)=>  TTA (L) | 1 | 773 | 258 | TCA (S) => TTA (L) | | 1 |
|  | 791 | 264 | CCC (P)=> CTC (L) | 1 | 791 | 264 | CCC (P) => CTC (L) | | 1 |
|  | 914 | 305 | TCA (S)=> TTA (L) | 1 | 914 | 305 | TCA (S) => TTA (L) | | 1 |
|  | 1,148 | 383 | TCA (S)=> TTA (L) | 1 | 1,148 | 383 | TCA (S) => TTA (L) | | 1 |
| *atpB* | 1,184 | 395 | TCA (S)=> TTA (L) | 1 | 1,184 | 395 | TCA (S) => TTA (L) | | 1 |
| *atpF* | 92 | 31 | CCA (P)=> CTA (L) | 0.86 | 92 | 31 | CCA (P) => CTA (L) | | 0.86 |
|  | 419 | 140 | GCC (A)=> GTC (V) | 0.86 | 419 | 140 | GCC (A) => GTC (V) | | 0.86 |
| *atpI* | 349 | 117 | CGG (R)=> TGG (W) | 1 | 349 | 117 | CGG (R) => TGG (W) | | 1 |
|  | 428 | 143 | CCC (P)=> CTC (L) | 1 | 428 | 143 | CCC (P) => CTC (L) | | 1 |
|  | 629 | 210 | TCA (S)=> TTA (L) | 1 | 629 | 210 | TCA (S)=> TTA (L) | | 1 |
| *ccsA* | 383 | 128 | ACA (T)=> ATA (I) | 0.86 | 383 | 128 | ACA (T) => ATA (I) | | 0.86 |
|  | 821 | 274 | TCA (S)=> TTA (L) | 1 | 821 | 274 | TCA (S) => TTA (L) | | 1 |
| *clpP* | 82 | 28 | CAT (H)=> TAT (Y) | 1 | 82 | 28 | CAT (H) => TAT (Y) | | 1 |
|  | 559 | 187 | CAT (H)=> TAT (Y) | 1 | 559 | 187 | CAT (H) => TAT (Y) | | 1 |
| *ndhA* | 50 | 17 | TCG (S)=> TTG (L) | 1 | 50 | 17 | TCG (S) => TTG (L) | | 1 |
|  | 395 | 132 | TCT (S)=> TTT (F) | 1 | 395 | 132 | TCT (S) => TTT (F) | | 1 |
|  | 476 | 159 | TCA (S)=> TTA (L) | 1 | 476 | 159 | TCA (S) => TTA (L) | | 1 |
|  | 566 | 189 | TCA (S)=> TTA (L) | 1 | 566 | 189 | TCA (S) => TTA (L) | | 1 |
|  | 1,073 | 358 | TCC (S)=> TTC (F) | 1 | 1,073 | 358 | TCC (S) => TTC (F) | | 1 |

**Table S3.** *Cont.*

| **Gene** | **Nucleotide**  **Position** | **Amino Acid**  **Position** | **Codon**  **Conversion** | **Score** | **Nucleotide**  **position** | **Amino Acid**  **Position** | **Codon**  **Conversion** | **Score** |
| --- | --- | --- | --- | --- | --- | --- | --- | --- |
|  | *Symplocarpus nipponicus* (Japan) | | | | *Symplocarpus nipponicus* (Korea) | | | |
| *ndhB* | 149 | 50 | TCA (S)=> TTA (L) | 1 | 149 | 50 | TCA (S) => TTA (L) | 1 |
|  | 446 | 149 | TCA (S)=> TTA (L) | 1 | 446 | 149 | TCA (S) => TTA (L) | 1 |
|  | 467 | 156 | CCA (P)=> CTA (L) | 1 | 467 | 156 | CCA (P) => CTA (L) | 1 |
|  | 542 | 181 | ACG (T)=> ATG (M) | 1 | 542 | 181 | ACG (T) => ATG (M) | 1 |
|  | 586 | 196 | CAT (H)=> TAT (Y) | 1 | 586 | 196 | CAT (H) => TAT (Y) | 1 |
|  | 704 | 235 | TCC (S)=> TTC (F) | 1 | 704 | 235 | TCC (S) => TTC (F) | 1 |
|  | 737 | 246 | CCA (P)=> CTA (L) | 1 | 737 | 246 | CCA (P) => CTA (L) | 1 |
|  | 830 | 277 | TCA (S)=> TTA (L) | 1 | 830 | 277 | TCA (S) => TTA (L) | 1 |
|  | 836 | 279 | TCA (S)=> TTA (L) | 1 | 836 | 279 | TCA (S) => TTA (L) | 1 |
|  | 1,102 | 368 | CGC (R)=> TGC (C) | 1 | 1,102 | 368 | CGC (R) => TGC (C) | 1 |
|  | 1,481 | 494 | CCA (P)=> CTA (L) | 1 | 1,481 | 494 | CCA (P) => CTA (L) | 1 |
| *ndhD* | 56 | 19 | ACG (T)=> ATG (M) | 1 | 56 | 19 | ACG (T) => ATG (M) | 1 |
|  | 113 | 38 | TCA (S)=> TTA (L) | 1 | 113 | 38 | TCA (S) => TTA (L) | 1 |
|  | 437 | 146 | TCA (S)=> TTA (L) | 1 | 437 | 146 | TCA (S) => TTA (L) | 1 |
|  | 602 | 201 | CCA (P)=> CTA (L) | 1 | 602 | 201 | CCA (P) => CTA (L) | 1 |
|  | 932 | 311 | TCA (S)=> TTA (L) | 1 | 932 | 311 | TCA (S) => TTA (L) | 1 |
|  | 1,001 | 334 | ACA (T)=> ATA (I) | 1 | 1,001 | 334 | ACA (T) => ATA (I) | 1 |
|  | 1,247 | 416 | TCA (S)=> TTA (L) | 0.8 | 1,247 | 416 | TCA (S) => TTA (L) | 0.8 |
|  | 1,352 | 451 | TCA (S)=> TTA (L) | 0.8 | 1,352 | 451 | TCA (S) => TTA (L) | 0.8 |
| *ndhF* | 62 | 21 | TCA (S)=> TTA (L) | 1 | 62 | 21 | TCA (S) => TTA (L) | 1 |
|  | 290 | 97 | TCA (S)=> TTA (L) | 1 | 290 | 97 | TCA (S) => TTA (L) | 1 |
|  | 392 | 131 | TCC (S)=> TTC (F) | 1 | 392 | 131 | TCC (S) => TTC (F) | 1 |
|  | 442 | 148 | CAT (H)=> TAT (Y) | 1 | 442 | 148 | CAT (H) => TAT (Y) | 1 |
|  | 632 | 211 | ACA (T)=> ATA (I) | 1 | 632 | 211 | ACA (T) => ATA (I) | 1 |
|  | 1460 | 487 | GCC (A) => GTC (V) | 0.8 | 1460 | 487 | GCC (A) => GTC (V) | 0.8 |
| *ndhG* | 314 | 105 | ACA (T)=> ATA (I) | - | 314 | 105 | ACA (T) => ATA (I) | 0.8 |
| *petB* | 418 | 140 | CGG (R)=> TGG (W) | 1 | 418 | 140 | CGG (R) => TGG (W) | 1 |
|  | 611 | 204 | TCA (S)=> TTA (L) | 1 | **-** | | | |
| *psbE* | 26 | 9 | ACA (T)=> ATA (I) | 0.86 | 214 | 72 | CCT (P) => TCT (S) | 1 |

**Table S3.** *Cont.*

| **Gene** | **Nucleotide**  **Position** | **Amino Acid**  **Position** | **Codon**  **Conversion** | **Score** | **Nucleotide**  **position** | **Amino Acid**  **Position** | **Codon**  **Conversion** | | **Score** |
| --- | --- | --- | --- | --- | --- | --- | --- | --- | --- |
|  | *Symplocarpus nipponicus* (Japan) | | | | *Symplocarpus nipponicus* (Korea) | | | | |
| *psbE* | 290 | 97 | CCT (P)=> CTT (L) | 1 | - | | | | |
| *psbF* | 77 | 26 | TCT (S)=> TTT (F) | 1 | 77 | 26 | | TCT (S) => TTT (F) | 1 |
| *rpl20* | 26 | 9 | ACA (T)=> ATA (I) | 0.86 | 26 | 9 | | ACA (T) => ATA (I) | 0.86 |
|  | 290 | 97 | CCT (P)=> CTT (L) | 1 | 290 | 97 | | CCT (P) => CTT (L) | 1 |
| *rpoA* | 830 | 277 | TCA (S)=> TTA (L) | 1 | 830 | 277 | | TCA (S) => TTA (L) | 1 |
| *rpoB* | 338 | 113 | TCT (S)=> TTT (F) | 1 | 338 | 113 | | TCT (S) => TTT (F) | 1 |
|  | 473 | 158 | TCA (S)=> TTA (L) | 0.86 | 473 | 158 | | TCA (S) => TTA (L) | 0.86 |
|  | 551 | 184 | TCA (S)=> TTA (L) | 1 | 551 | 184 | | TCA (S) => TTA (L) | 1 |
|  | 566 | 189 | TCG (S)=> TTG (L) | 1 | 566 | 189 | | TCG (S) => TTG (L) | 1 |
|  | 623 | 208 | CCG (P)=> CTG (L) | 0.86 | 623 | 208 | | CCG (P) => CTG (L) | 0.86 |
|  | 2,432 | 811 | TCA (S)=> TTA (L) | 0.86 | 2,432 | 811 | | TCA (S) => TTA (L) | 0.86 |
| *rpoC1* | 62 | 21 | CCA (P)=> CTA (L) | 1 | 62 | 21 | | CCA (P) => CTA (L) | 1 |
|  | 203 | 68 | TCT (S)=> TTT (F) | 1 | 203 | 68 | | TCT (S) => TTT (F) | 1 |
|  | 638 | 213 | TCG (S)=> TTG (L) | 1 | 638 | 213 | | TCG (S) => TTG (L) | 1 |
| *rpoC2* | 1,447 | 483 | CCT (P)=> TCT (S) | 1 | 1,447 | 483 | | CCT (P) => TCT (S) | 1 |
|  | 1,771 | 591 | CGT (R)=> TGT (C) | 0.86 | 1,771 | 591 | | CGT (R) => TGT (C) | 0.86 |
|  | 2,284 | 762 | CGG (R)=> TGG (W) | 1 | 2,284 | 762 | | CGG (R) => TGG (W) | 1 |
|  | 2,318 | 773 | TCG (S)=> TTG (L) | 1 | 2,318 | 773 | | TCG (S) => TTG (L) | 1 |
|  | 3,707 | 1,236 | TCA (S)=> TTA (L) | 0.86 | 3,707 | 1,236 | | TCA (S) => TTA (L) | 0.86 |
| *rps2* | 248 | 83 | TCA (S)=> TTA (L) | 1 | 248 | 83 | | TCA (S) => TTA (L) | 1 |
| *rps8* | 182 | 61 | TCA (S)=> TTA (L) | 0.86 | 182 | 61 | | TCA (S) => TTA (L) | 0.86 |
| *rps14* | 80 | 27 | TCA (S)=> TTA (L) | 1 | 80 | 27 | | TCA (S) => TTA (L) | 1 |
|  | 149 | 50 | CCA (P)=> CTA (L) | 1 | 149 | 50 | | CCA (P) => CTA (L) | 1 |
| *rps16* | 143 | 48 | TCA (S)=> TTA (L) | 1 | 143 | 48 | | TCA (S) => TTA (L) | 1 |
| *ycf3* | 44 | 15 | TCT (S)=> TTT (F) | 1 | 44 | 15 | | TCT (S) => TTT (F) | 1 |
|  | 185 | 62 | ACG (T)=> ATG (M) | 1 | 185 | 62 | | ACG (T) => ATG (M) | 1 |
|  | 191 | 64 | CCA (P)=> CTA (L) | 1 | 191 | 64 | | CCA (P) => CTA (L) | 1 |
|  | 407 | 136 | TCC (S)=> TTC (F) | 1 | 407 | 136 | | TCC (S) => TTC (F) | 1 |
