# Supplementary material for: Comparison of Whole Plastome Sequences between Thermogenic Skunk Cabbage Symplocarpus renifolius and Nonthermogenic S. nipponicus (Orontioideae; Araceae) in East Asia
Source: Int J Mol Sci. 2019 Sep 20;20(19):4678. doi: 10.3390/ijms20194678 (PMC6801674; doi:10.3390/ijms20194678)
Supplement: Supplementary file 1 [file ijms-20-04678-s001.zip › Table S4.docx]

**Table S4.** Repeat sequences and their distribution in the *Symplocarpus nipponicus* chloroplast genome in Japan.

| cpSSR ID | Repeat Motif | Length (bp) | Start | End | Region | Annotation |
| --- | --- | --- | --- | --- | --- | --- |
| 1 | (TA) 4 | 8 | 1,519 | 1,526 | LSC |  |
| 2 | (AT) 23 | 46 | 5,338 | 5,383 | LSC | *rps16* intron |
| 3 | (AT) 7 | 14 | 5,438 | 5,451 | LSC | *rps16* intron |
| 4 | (T) 13 | 13 | 6,898 | 6,910 | LSC |  |
| 5 | (A) 10 | 10 | 7,994 | 8,003 | LSC |  |
| 6 | (T) 10 | 10 | 8,093 | 8,102 | LSC |  |
| 7 | (A) 10 | 10 | 8,136 | 8,145 | LSC |  |
| 8 | (T) 16 | 16 | 8,364 | 8,379 | LSC |  |
| 9 | (GA) 4 | 8 | 8,442 | 8,449 | LSC | *trnS* gene |
| 10 | (T) 11 | 11 | 9,026 | 9,036 | LSC |  |
| 11 | (A) 12 | 12 | 9,320 | 9,331 | LSC |  |
| 12 | (A) 11 | 11 | 12,328 | 12,338 | LSC |  |
| 13 | (A) 10 | 10 | 13,516 | 13,525 | LSC | *atpF* intron |
| 14 | (A) 10 | 10 | 14,137 | 14,146 | LSC |  |
| 15 | (T) 15 | 15 | 15,124 | 15,138 | LSC |  |
| 16 | (A) 12 | 12 | 15,275 | 15,286 | LSC |  |
| 17 | (TA) 8 | 16 | 16,184 | 16,199 | LSC |  |
| 18 | (TTA) 4 | 12 | 16,277 | 16,288 | LSC |  |
| 19 | (T) 11 | 11 | 19,229 | 19,239 | LSC |  |
| 20 | (AT) 4 | 8 | 20,512 | 20,519 | LSC | *rpoC2* gene |
| 21 | (AT) 5 | 10 | 20,602 | 20,611 | LSC | *rpoC2* gene |
| 22 | (C) 10 | 10 | 23,168 | 23,177 | LSC | *rpoC1* intron |
| 23 | (T) 12 | 12 | 23,679 | 23,690 | LSC | *rpoC1* intron |
| 24 | (AT) 4 | 8 | 27,676 | 27,683 | LSC |  |
| 25 | (A) 12 | 12 | 28,347 | 28,358 | LSC |  |
| 26 | (T) 12 | 12 | 28,387 | 28,398 | LSC |  |
| 27 | (TG) 4 | 8 | 29,068 | 29,075 | LSC |  |
| 28 | (A) 10 | 10 | 29,521 | 29,530 | LSC |  |
| 29 | (A) 11 | 11 | 29,675 | 29,685 | LSC |  |
| 30 | (A) 12 | 12 | 29,762 | 29,773 | LSC |  |
| 31 | (T) 10 | 10 | 30,436 | 30,445 | LSC |  |
| 32 | (T) 13 | 13 | 31,036 | 31,048 | LSC |  |
| 33 | (T) 11 | 11 | 31,595 | 31,605 | LSC |  |
| 34 | (T) 10 | 10 | 32,311 | 32,320 | LSC |  |
| 35 | (AAT) 5 | 15 | 33,678 | 33,692 | LSC |  |
| 36 | (TA) 6 | 12 | 36,522 | 36,533 | LSC |  |
| 37 | (GA) 4 | 8 | 36,645 | 36,652 | LSC | *trnS* gene |
| 38 | (T) 10 | 10 | 37,387 | 37,396 | LSC |  |
| 39 | (T) 10 | 10 | 37,454 | 37,463 | LSC |  |
| 40 | (AT) 5 | 10 | 43,481 | 43,490 | LSC |  |
| 41 | (T) 10 | 10 | 45,533 | 45,542 | LSC | *ycf3* intron |
| 42 | (A) 12 | 12 | 45,592 | 45,603 | LSC | *ycf3* intron |
| 43 | (TA) 4 | 8 | 47,079 | 47,086 | LSC |  |
| 44 | (AT) 15 | 30 | 47,539 | 47,568 | LSC |  |
| 45 | (A) 13 | 13 | 47,614 | 47,626 | LSC |  |
| 46 | (AT) 10 | 20 | 47,654 | 47,673 | LSC |  |
| 47 | (T) 11 | 11 | 47,910 | 47,920 | LSC |  |
| 48 | (A) 10 | 10 | 48,198 | 48,207 | LSC | *trnL* intron |
| 49 | (TA) 4 | 8 | 48,338 | 48,345 | LSC | *trnL* intron |
| 50 | (AT) 5 | 10 | 48,410 | 48,419 | LSC | *trnL* intron |
| 51 | (A) 13 | 13 | 48,557 | 48,569 | LSC | *trnL* intron |
| 52 | (AG) 4 | 8 | 48,591 | 48,598 | LSC | *trnL* intron |
| 53 | (AT) 4 | 8 | 49,083 | 49,090 | LSC |  |
| 54 | (T) 10 | 10 | 51,537 | 51,546 | LSC |  |
| 55 | (AT) 10 | 20 | 51,724 | 51,743 | LSC |  |
| 56 | (T) 10 | 10 | 51,770 | 51,779 | LSC |  |
| 57 | (T) 10 | 10 | 53,543 | 53,552 | LSC |  |
| 58 | (T) 10 | 10 | 53,698 | 53,707 | LSC |  |
| 59 | (TA) 10 | 20 | 56,005 | 56,024 | LSC |  |
| 60 | (T) 11 | 11 | 56,403 | 56,413 | LSC |  |
| 61 | (GA) 4 | 8 | 56,969 | 56,976 | LSC | *rbcL* gene |
| 62 | (TA) 4 | 8 | 58,135 | 58,142 | LSC |  |
| 63 | (TTATA) 6 | 30 | 58,349 | 58,378 | LSC |  |
| 64 | (AT) 5 | 10 | 58,534 | 58,543 | LSC |  |
| 65 | (TG) 4 | 8 | 59,829 | 59,836 | LSC | *accD* gene |
| 66 | (A) 11 | 11 | 60,208 | 60,218 | LSC | *accD* gene |
| 67 | (T) 10 | 10 | 62,871 | 62,880 | LSC |  |
| 68 | (A) 12 | 12 | 63,096 | 63,107 | LSC |  |
| 69 | (TC) 5 | 10 | 63,164 | 63,173 | LSC |  |
| 70 | (T) 10 | 10 | 65,657 | 65,666 | LSC |  |
| 71 | (AT) 4 | 8 | 68,871 | 68,878 | LSC |  |
| 72 | (A) 10 | 10 | 70,355 | 70,364 | LSC |  |
| 73 | (TA) 4 | 8 | 71,803 | 71,810 | LSC | *clpP* intron |
| 74 | (AT) 5 | 10 | 71,917 | 71,926 | LSC | *clpP* intron |
| 75 | (A) 11 | 11 | 72,307 | 72,317 | LSC | *clpP* intron |
| 76 | (TTA) 5 | 15 | 72,619 | 72,633 | LSC | *clpP* intron |
| 77 | (A) 10 | 10 | 73,278 | 73,287 | LSC | *clpP* intron |
| 78 | (A) 11 | 11 | 73,408 | 73,418 | LSC | *clpP* intron |
| 79 | (T) 11 | 11 | 73,621 | 73,631 | LSC | *clpP* intron |
| 80 | (TA) 10 | 20 | 74,451 | 74,470 | LSC |  |
| 81 | (A) 12 | 12 | 76,905 | 76,916 | LSC |  |
| 82 | (A) 11 | 11 | 80,065 | 80,075 | LSC |  |
| 83 | (A) 17 | 17 | 81,952 | 81,968 | LSC |  |
| 84 | (AT) 6 | 12 | 83,959 | 83,970 | LSC | *rpl16* intron |
| 85 | (GA) 4 | 8 | 89,645 | 89,652 | IRb | *ycf2* gene |
| 86 | (GA) 4 | 8 | 91,851 | 91,858 | IRb | *ycf2* gene |
| 87 | (TA) 4 | 8 | 95,234 | 95,241 | IRb | *ycf2* gene |
| 88 | (AG) 4 | 8 | 97,554 | 97,561 | IRb | *ndhB* exon |
| 89 | (T) 10 | 10 | 101,460 | 101,469 | IRb |  |
| 90 | (T) 10 | 10 | 105,502 | 105,511 | IRb |  |
| 91 | (CT) 4 | 8 | 108,839 | 108,846 | IRb |  |
| 92 | (A) 13 | 13 | 110,542 | 110,554 | IRb | *trnI* gene |
| 93 | (TA) 4 | 8 | 111,741 | 111,748 | IRb |  |
| 94 | (TTA) 4 | 12 | 112,332 | 112,343 | SSC |  |
| 95 | (AT) 5 | 10 | 112,568 | 112,577 | SSC |  |
| 96 | (AAT) 4 | 12 | 112,655 | 112,666 | SSC |  |
| 97 | (T) 11 | 11 | 114,933 | 114,943 | SSC |  |
| 98 | (T) 12 | 12 | 115,747 | 115,758 | SSC |  |
| 99 | (AT) 5 | 10 | 115,813 | 115822 | SSC |  |
| 100 | (AT) 4 | 8 | 115,947 | 115,954 | SSC |  |
| 101 | (A) 11 | 11 | 116,523 | 116,533 | SSC |  |
| 102 | (A) 10 | 10 | 116,998 | 117,007 | SSC |  |
| 103 | (A) 12 | 12 | 120,884 | 120,895 | SSC |  |
| 104 | (T) 14 | 14 | 121,542 | 121,555 | SSC |  |
| 105 | (G) 14 | 14 | 121,622 | 121,635 | SSC |  |
| 106 | (T) 10 | 10 | 124,210 | 124219 | SSC |  |
| 107 | (A) 13 | 13 | 124,323 | 124,335 | SSC |  |
| 108 | (A) 10 | 10 | 124,813 | 124,822 | SSC | *ndhA* intron |
| 109 | (CT) 4 | 8 | 126,228 | 126,235 | SSC | *ndhH* gene |
| 110 | (T) 11 | 11 | 127,039 | 127,049 | SSC |  |
| 111 | (T) 11 | 11 | 128,301 | 128,311 | SSC | *ycf1* gene |
| 112 | (T) 12 | 12 | 128,580 | 128,591 | SSC | *ycf1* gene |
| 113 | (T) 10 | 10 | 129,087 | 129,096 | SSC | *ycf1* gene |
| 114 | (T) 11 | 11 | 130,178 | 130,188 | SSC | *ycf1* gene |
| 115 | (A) 12 | 12 | 130,273 | 130,284 | SSC | *ycf1* gene |
| 116 | (AT) 4 | 8 | 133,007 | 133,014 | IRa |  |
| 117 | (T) 13 | 13 | 134,202 | 134,214 | IRa |  |
| 118 | (AG) 4 | 8 | 135,910 | 135,917 | IRa | *23S rRNA* gene |
| 119 | (A) 10 | 10 | 139,245 | 139,254 | IRa | *trnI* gene |
| 120 | (A) 10 | 10 | 143,287 | 143,296 | IRa |  |
| 121 | (CT) 4 | 8 | 147,195 | 147,202 | IRa | *rps12* exon |
| 122 | (TA) 4 | 8 | 149,515 | 149,522 | IRa |  |
| 123 | (CT) 4 | 8 | 152,897 | 152,904 | IRa | *ycf2* gene |
| 124 | (TC) 4 | 8 | 155,104 | 155,111 | IRa | *ycf2* gene |
| 125 | C | 20 | 7,287 | 7,306 | LSC |  |
| 126 | C | 35 | 13,784 | 13,818 | LSC |  |
| 127 | C | 21 | 23,626 | 23,646 | LSC |  |
| 128 | C | 29 | 32,966 | 32,994 | LSC |  |
| 129 | C | 37 | 47,469 | 47,505 | LSC |  |
| 130 | C | 39 | 79,885 | 79,923 | LSC |  |
| 131 | C | 20 | 88,631 | 88,650 | IRb |  |
| 132 | C | 32 | 96,788 | 96,819 | IRb |  |
| 133 | C | 19 | 120,928 | 120,946 | SSC |  |
| 134 | C | 31 | 147,937 | 147,967 | IRa |  |
| 135 | C | 20 | 156,106 | 156,125 | IRa |  |

Notes: 1-124 represents unique consensus SSRs. 125-135 represents compound repeats as C. Also, total of 11 SSRs (out of 146 copies) identified as compound formation.
