# Supplementary material for: Comparison of Whole Plastome Sequences between Thermogenic Skunk Cabbage Symplocarpus renifolius and Nonthermogenic S. nipponicus (Orontioideae; Araceae) in East Asia
Source: Int J Mol Sci. 2019 Sep 20;20(19):4678. doi: 10.3390/ijms20194678 (PMC6801674; doi:10.3390/ijms20194678)
Supplement: Supplementary file 1 [file ijms-20-04678-s001.zip › Table S5.docx]

**Table S5.** Repeat sequences and their distribution in the *Symplocarpus nipponicus* chloroplast genome in Korea.

| cpSSR ID | Repeat Motif | Length (bp) | Start | End | Region | Annotation |
| --- | --- | --- | --- | --- | --- | --- |
| 1 | (A) 10 | 10 | 178 | 187 | LSC |  |
| 2 | (TA) 4 | 8 | 1,520 | 1,527 | LSC |  |
| 3 | (A) 10 | 10 | 3,756 | 3,765 | LSC | *trnK* intron |
| 4 | (AT )8 | 16 | 5,344 | 5,359 | LSC | *rps16* intron |
| 5 | (T) 13 | 13 | 6,872 | 6,884 | LSC |  |
| 6 | (A) 10 | 10 | 7,968 | 7,977 | LSC |  |
| 7 | (A) 10 | 10 | 8,109 | 8,118 | LSC |  |
| 8 | (T) 11 | 11 | 8,999 | 9,009 | LSC |  |
| 9 | (A) 11 | 11 | 9,293 | 9,303 | LSC |  |
| 10 | (A) 10 | 10 | 12,300 | 12,309 | LSC |  |
| 11 | (A) 10 | 10 | 13,487 | 13,496 | LSC | *atpF* intron |
| 12 | (A) 10 | 10 | 14,111 | 14,120 | LSC |  |
| 13 | (T) 14 | 14 | 15,101 | 15,114 | LSC |  |
| 14 | (A) 11 | 11 | 15,251 | 15,261 | LSC |  |
| 15 | (T) 11 | 11 | 19,212 | 19,222 | LSC | *rpoC2* gene |
| 16 | (AT) 4 | 8 | 27,659 | 27,666 | LSC |  |
| 17 | (TG) 4 | 8 | 29,050 | 29,057 | LSC |  |
| 18 | (A) 10 | 10 | 29,503 | 29,512 | LSC |  |
| 19 | (T) 10 | 10 | 30,415 | 30,424 | LSC |  |
| 20 | (T) 12 | 12 | 31,015 | 31,026 | LSC |  |
| 21 | (T) 13 | 13 | 31,573 | 31,585 | LSC |  |
| 22 | (T) 10 | 10 | 32,416 | 32,425 | LSC |  |
| 23 | (AAT) 7 | 21 | 33,783 | 33,803 | LSC |  |
| 24 | (T) 10 | 10 | 33,905 | 33,914 | LSC |  |
| 25 | (TA) 6 | 12 | 36,634 | 36,645 | LSC |  |
| 26 | (GA) 4 | 8 | 36,757 | 36,764 | LSC |  |
| 27 | (AT) 5 | 10 | 43,589 | 43,598 | LSC |  |
| 28 | (TA) 4 | 8 | 47,189 | 47,196 | LSC |  |
| 29 | (T) 10 | 10 | 48,015 | 48,024 | LSC |  |
| 30 | (A) 11 | 11 | 48,302 | 48,312 | LSC | *trnF* intron |
| 31 | (AT) 4 | 8 | 49,192 | 49,199 | LSC |  |
| 32 | (T) 10 | 10 | 51,646 | 51,655 | LSC |  |
| 33 | (T) 10 | 10 | 53,649 | 53,658 | LSC |  |
| 34 | (TA) 11 | 22 | 56,110 | 56,131 | LSC |  |
| 35 | (T) 11 | 11 | 56,510 | 56,520 | LSC |  |
| 36 | (GA) 4 | 8 | 57,076 | 57,083 | LSC | *rbcL* gene |
| 37 | (TA) 4 | 8 | 58,242 | 58,249 | LSC |  |
| 38 | (AT) 5 | 10 | 58,626 | 58,635 | LSC |  |
| 39 | (TG) 4 | 8 | 59,921 | 59,928 | LSC | *accD* gene |
| 40 | (A) 10 | 10 | 60,300 | 60,309 | LSC |  |
| 41 | (T) 10 | 10 | 63,017 | 63,026 | LSC |  |
| 42 | (T) 11 | 11 | 65,802 | 65,812 | LSC |  |
| 43 | (T) 10 | 10 | 67,551 | 67,560 | LSC |  |
| 44 | (AT) 4 | 8 | 69,018 | 69,025 | LSC |  |
| 45 | (A) 10 | 10 | 70,502 | 70,511 | LSC |  |
| 46 | (TA) 4 | 8 | 71,950 | 71,957 | LSC |  |
| 47 | (AT) 5 | 10 | 72,064 | 72,073 | LSC |  |
| 48 | (A) 11 | 11 | 72,454 | 72,464 | LSC | *clpP* intron2 |
| 49 | (TTA) 5 | 15 | 72,766 | 72,780 | LSC | *clpP* intron2 |
| 50 | (A) 12 | 12 | 73,425 | 73,436 | LSC | *clpP* intron 1 |
| 51 | (A) 10 | 10 | 73,557 | 73,566 | LSC | *clpP* intron 1 |
| 52 | (T) 14 | 14 | 73,769 | 73,782 | LSC | *clpP* intron 1 |
| 53 | (TA) 15 | 30 | 74,602 | 74,631 | LSC |  |
| 54 | (A) 12 | 12 | 77,066 | 77,077 | LSC |  |
| 55 | (AT) 14 | 28 | 80,046 | 80,073 | LSC |  |
| 56 | (A) 12 | 12 | 80,215 | 80,226 | LSC |  |
| 57 | (A) 19 | 19 | 82,103 | 82,121 | LSC |  |
| 58 | (T) 10 | 10 | 82,931 | 82,940 | LSC |  |
| 59 | (AT) 6 | 12 | 84,113 | 84,124 | LSC | *rpl16* intron |
| 60 | (GA) 4 | 8 | 89,807 | 89,814 | IRb | *ycf2* gene |
| 61 | (GA) 4 | 8 | 92,013 | 92,020 | IRb | *ycf2* gene |
| 62 | (TA) 4 | 8 | 95,396 | 95,403 | IRb | *ycf2* gene |
| 63 | (AG) 4 | 8 | 97,716 | 97,723 | IRb | *ndhB* exon |
| 64 | (T) 10 | 10 | 101,622 | 101,631 | IRb |  |
| 65 | (T) 10 | 10 | 105,664 | 105,673 | IRb | *trnI* intron |
| 66 | (CT) 4 | 8 | 109,001 | 109,008 | IRb | *23S rRNA* gene |
| 67 | (A) 12 | 12 | 110,704 | 110,715 | IRb | *23S rRNA* gene |
| 68 | (TA) 4 | 8 | 111,902 | 111,909 | IRb |  |
| 69 | (T) 10 | 10 | 115,096 | 115,105 | SSC |  |
| 70 | (AT) 4 | 8 | 116,134 | 116,141 | SSC |  |
| 71 | (A) 10 | 10 | 117,183 | 117,192 | SSC |  |
| 72 | (A) 10 | 10 | 118,643 | 118,652 | SSC |  |
| 73 | (A) 11 | 11 | 124,510 | 124,520 | SSC | *ndhA* intron1 |
| 74 | (A) 10 | 10 | 124,998 | 125,007 | SSC | *ndhA* intron1 |
| 75 | (CT) 4 | 8 | 126,413 | 126,420 | SSC | *ndhH* gene |
| 76 | (T) 12 | 12 | 127,224 | 127,235 | SSC |  |
| 77 | (T) 11 | 11 | 128,494 | 128,504 | SSC | *ycf1* gene |
| 78 | (T) 12 | 12 | 128,773 | 128,784 | SSC | *ycf1* gene |
| 79 | (T) 10 | 10 | 129,280 | 129,289 | SSC | *ycf1* gene |
| 80 | (AT) 4 | 8 | 133,194 | 133,201 | IRa |  |
| 81 | (T) 12 | 12 | 134,389 | 134,400 | IRa |  |
| 82 | (AG) 4 | 8 | 136,096 | 136,103 | IRa | *23S rRNA* gene |
| 83 | (A) 10 | 10 | 139,431 | 139,440 | IRa | *trnI* intron |
| 84 | (A) 10 | 10 | 143,473 | 143,482 | IRa |  |
| 85 | (CT) 4 | 8 | 147,381 | 147,388 | IRa | *ndhB* exon2 |
| 86 | (TA) 4 | 8 | 149,701 | 149,708 | IRa | *ycf2* gene |
| 87 | (CT) 4 | 8 | 153,083 | 153,090 | IRa | *ycf2* gene |
| 88 | (TC) 4 | 8 | 155,290 | 155,297 | IRa | *ycf2* gene |
| 89 | C | 29 | 33,071 | 33,099 | LSC |  |
| 90 | C | 20 | 7,261 | 7,280 | LSC |  |
| 91 | C | 86 | 8,337 | 8,422 | LSC |  |
| 92 | C | 38 | 13,755 | 13,792 | LSC |  |
| 93 | C | 113 | 16,159 | 16,271 | LSC |  |
| 94 | C | 100 | 20,495 | 20,594 | LSC |  |
| 95 | C | 66 | 23,608 | 23,673 | LSC |  |
| 96 | C | 51 | 28,330 | 28,380 | LSC |  |
| 97 | C | 96 | 29,657 | 29,752 | LSC |  |
| 98 | C | 73 | 37,499 | 37,571 | LSC |  |
| 99 | C | 73 | 45,641 | 45,713 | LSC |  |
| 100 | C | 172 | 47,607 | 47,778 | LSC |  |
| 101 | C | 87 | 48,443 | 48,529 | LSC |  |
| 102 | C | 41 | 48,667 | 48,707 | LSC |  |
| 103 | C | 53 | 51,833 | 51,885 | LSC |  |
| 104 | C | 77 | 63,242 | 63,318 | LSC |  |
| 105 | C | 20 | 88,793 | 88,812 | IRb |  |
| 106 | C | 32 | 96,950 | 96,981 | IRb |  |
| 107 | C | 176 | 112,404 | 112,579 | SSC |  |
| 108 | C | 102 | 112,728 | 112,829 | SSC |  |
| 109 | C | 77 | 115,933 | 116,009 | SSC |  |
| 110 | C | 65 | 121,071 | 121,135 | SSC |  |
| 111 | C | 93 | 121,731 | 121,823 | SSC |  |
| 112 | C | 107 | 130,371 | 130,477 | SSC |  |
| 113 | C | 31 | 148,123 | 148,153 | IRa |  |
| 114 | C | 20 | 156,292 | 156,311 | IRa |  |

Notes: 1-88 represents unique consensus SSRs. 89-114 represents compound repeats as C. Also, total of 33 SSRs (out of 147 copies) identified as compound formation.
